# Genome-wide characterization of the PPR gene family and its potential roles in stress responses and chloroplast RNA editing in *Brassica rapa*

**DOI:** 10.3389/fpls.2026.1860005

**Published:** 2026-06-15

**Authors:** Jinghong Zhang, Min Chen, Ju Zhang, Fangyan Hu, Kexin Su, Yina Zhi, Yangshuo Huang, Junya Hu, Zhenmeng Tian, Qiurui Yang, Wenjuan Li, Kedong Xu, Fei Zhou

**Affiliations:** 1 Key Laboratory of Plant Genetics and Molecular Breeding, Zhoukou Normal University, Zhoukou, China; 2 School of Computer Science and Technology, Zhoukou Normal University, Zhoukou, China; 3 Henan Key Laboratory of Crop Molecular Breeding and Bioreactor, Zhoukou Normal University, Zhoukou, China; 4 National Key Laboratory of Crop Genetic Improvement, Huazhong Agricultural University, Wuhan, China

**Keywords:** *Brassica rapa*, expression patterns, PPR protein, RNA editing, stress responses

## Abstract

**Introduction:**

Pentatricopeptide repeat proteins are a large family of RNA-binding proteins that play essential roles in post-transcriptional regulation within plant organelles. However, a systematic understanding of their evolutionary expansion and functional relevance in *Brassica rapa* remains limited. This study identified and characterized the PPR gene family in *B. rapa* and investigated their potential roles in stress responses and chloroplast RNA editing.

**Methods:**

Using the *B. rapa* Chiifu v4.0 genome assembly, we performed a genome-wide identification and characterization of PPR genes. Phylogenetic relationships, gene structures, duplication patterns, chromosomal distribution, subcellular localization, and cis-regulatory elements were analyzed. Tissue-specific expression patterns were investigated using publicly available RNA-seq datasets and qRT-PCR validation, while stress-associated transcriptional responses and organellar RNA editing profiles were analyzed using public RNA-seq datasets.

**Results:**

A genome-wide analysis identified 493 PPR genes, classified into P and PLS subfamilies, with uneven chromosomal distribution and expansion mainly driven by dispersed and whole-genome duplication events. Furthermore, subcellular localization prediction indicated that most PPR proteins are targeted to mitochondria and chloroplasts, consistent with their roles in organellar gene regulation. In addition, Gene Ontology enrichment analysis suggested potential associations of PPR proteins with RNA processing and RNA editing pathways. Moreover, promoter analysis identified numerous stress-responsive cis-acting elements, indicating that PPR genes may participate in transcriptional responses under environmental stress conditions. Meanwhile, expression profiling based on publicly available RNA-seq datasets revealed tissue-preferential expression patterns and stress-associated transcriptional changes under drought, heat, and immune elicitor treatments, with some PPR genes showing altered expression across multiple stress conditions. Chloroplast RNA editing analysis based on heat-stress RNA-seq datasets revealed dynamic and site-specific changes in editing efficiency. Several editing sites, including *cemA*, *psbZ*, and *ndhD*, showed relatively higher editing levels in the heat-tolerant line than in the heat-sensitive line. In contrast, prolonged heat stress was associated with reduced editing efficiency at multiple sites such as *atpF*, *rpoB*, *rps14*, and *clpP*.

**Discussion:**

Collectively, this study provides a comprehensive overview of PPR genes in *B. rapa* and identifies candidate PPR genes and stress-associated RNA editing events that may be relevant to stress-responsive regulation.

## 1 Introduction

Pentatricopeptide repeat (PPR) proteins constitute one of the largest protein families in land plants and are characterized by tandem arrays of a degenerate 35–amino acid motif (Small and Peeters, 2000). Based on motif composition and C-terminal domain features, PPR proteins are generally classified into two major subfamilies: the P-type, which contains only canonical P motifs, and the PLS-type, which is composed of P, L, and S motifs and often harbors additional E, E+, or DYW domains (Lurin et al., 2004). PPR proteins predominantly localize to mitochondria and chloroplasts, where they play essential roles in post-transcriptional regulation of organellar gene expression (Barkan and Small, 2014; Manna, 2015). Functionally, P-type PPR proteins are mainly involved in RNA stabilization and splicing, whereas PLS-type proteins are primarily associated with site-specific RNA editing, particularly cytidine-to-uridine (C-to-U) conversion in plant organelles (Barkan and Small, 2014). Through these functions, PPR proteins are indispensable for organelle biogenesis, photosynthesis, respiration, and overall plant development. Loss-of-function mutations in PPR genes often results in a range of developmental defects, including reduced seed germination, stunted seedling growth, abnormal leaf and flower morphology, decreased pollen viability, and lowered seed yield (Schmitz-Linneweber and Small, 2008; Barkan and Small, 2014; Li et al., 2025; Lee, 2026).

The PPR gene family are remarkably expanded in land plant, with most species encoding hundreds of PPR proteins. This expansion, together with extensive functional diversification, represents a key evolutionary innovation that enables fine-tuned regulation of organellar gene expression in response to developmental and environmental cues (Gutmann et al., 2020; Wang et al., 2021; Lee, 2026).

In recent years, genome-wide identification and characterization of PPR gene families have been conducted in several plant species, including *Arabidopsis thaliana*, rice (*Oryza sativa*), poplar (*Populus trichocarpa*), maize (*Zea mays*), and cotton (*Gossypium* spp.) (Lurin et al., 2004; Chen et al., 2018a, b; Xing et al., 2018; Zhao et al., 2018). These studies revealed extensive lineage-specific expansion of PPR genes, largely driven by dispersed duplications as well as whole-genome duplication (WGD) or segmental duplication events. In addition to their well-established roles in organelle RNA metabolism, accumulating evidence suggests that PPR proteins also participate in plant responses to abiotic and biotic stresses, including drought, cold, salinity, and pathogen attack (Chen et al., 2018a).

*Brassica rapa* is an important vegetable and oilseed crop and serves as a model for studying genome evolution in the Brassicaceae family. Its genome has undergone a whole-genome triplication (WGT) event, resulting in extensive gene duplication and functional diversification (Wang et al., 2011). Although the *B. rapa* genome has been well annotated, a comprehensive and systematic analysis of the PPR gene family in this species is still lacking. In particular, the evolutionary expansion patterns, structural characteristics, tissue-specific expression profiles, and stress-responsive behaviors of *B. rapa* PPR genes remain poorly understood.

In this study, we performed a genome-wide identification and characterization of the PPR gene family in *B. rapa* based on the Chiifu v4.0 genome assembly (Zhang et al., 2023b). We systematically analyzed their chromosomal distribution, gene structure, phylogenetic relationships, duplication patterns, and syntenic relationships. Furthermore, subcellular localization was predicted and functional enrichment was assessed using Gene Ontology annotations. To further explore their potential biological roles, we investigated tissue-preferential expression patterns and stress-associated transcriptional changes using publicly available RNA-seq datasets, including drought, heat and immunity elicitor treatments. In addition, cis-acting regulatory elements in the promoter regions of PPR genes were analyzed to elucidate potential transcriptional regulatory mechanisms. Collectively, this study provides a comprehensive resource for understanding the evolution and functional diversification of PPR genes in *B. rapa*, and provide valuable resources for future functional studies on their roles in plant development and stress adaptation.

## 2 Materials and methods

### 2.1 Identification of PPR genes in *B. rapa*

To identify PPR genes in *B. rapa* genome, protein sequence of *B. rapa* genome assembly of *B. rapa* Chiifu v4.0 (Zhang et al., 2023b) was used to predict motif using interproscan (version 5.71-102.0, default parameters) (Jones et al., 2014). Protein sequence with PPR motif PF01535 were submitted to the PPR database (https://ppr.plantenergy.uwa.edu.au/ppr/) to predict PPR motif number and other motifs (Cheng et al., 2016). Proteins with more than one PPR motif were kept as final PPR proteins. The final set of PPR proteins was then classified into either P-type or PLS-type based on the presence or absence of specific amino acid motifs (PLS, E1, E2, E+, and DYW).

### 2.2 Subcellular prediction and function enrichment analysis

Subcellular localization of PPR proteins was predicted using the online software ProtComp v9.0 (http://www.softberry.com/ last accessed on January 22, 2026).

eggNOG-mapper (version 2.1.12) was used to annotate PPR proteins and assign Gene Ontology (GO) terms, with parameters set to --query_cover 80 and --subject_cover 80 (Cantalapiedra et al., 2021). GO enrichment analysis was subsequently performed using the clusterProfiler R package (version 4.14.0) (Wu et al., 2021) based on customized GO annotation files generated by eggNOG-mapper.

### 2.3 Phylogenetic and synteny analysis

All *B. rapa* PPR protein sequences were submitted to EMBL-EBI Clustal Omega (https://www.ebi.ac.uk/jdispatcher/msa/clustalo) for multiple sequence alignment (Madeira et al., 2024). The same tool also generated a phylogenetic tree using PhyloTree.js (Shank et al., 2018). The resulting phylogenetic tree was visualized using the ggtree R package (Yu et al., 2017).

To analyze gene duplication and synteny, all *B. rapa* protein sequences were aligned against themselves using BLASTP, collinear blocks were identified using MCScanX (version 1.0.0) (Wang et al., 2012). Duplication events of PPR genes were classified using the duplicate_gene_classifier program implemented in MCScanX. All syntenic gene pairs and the chromosomal distribution of PPR genes were visualized using Circos (Krzywinski et al., 2009) and the chromoMap R package (Anand and Rodriguez Lopez, 2022), respectively.

Genome−wide collinearity between *Arabidopsis* and *Brassica rapa* was analyzed using the JCVI toolkit (version 1.6.5) (Tang et al., 2024). Gene coordinates were extracted from GFF files and converted to BED format using the jcvi.formats.gff bed module. Orthologous gene pairs and syntenic blocks were identified with the MCscan algorithm via jcvi.compara.catalog ortholog using coding sequences. A syntenic block containing the PPR gene family was extracted from the resulting anchors file. The synteny plot was generated with jcvi.graphics.synteny using the extracted block, a merged BED file of both species, and a custom layout file.

### 2.4 Prediction of cis-regulatory elements

To identify cis-acting regulatory elements in PPR gene promoters, 2-kb upstream sequences from the ATG start codon were extracted and analyzed using the PlantCARE database (http://bioinformatics.psb.ugent.be/webtools/plantcare/html/) (Lescot et al., 2002). The resulting PlantCARE annotations were further processed using the R package BioVisSeq (Zhao et al., 2025) and visualized with pheatmap (Kolde and Kolde, 2015).

### 2.5 Expression profiling of *B. rapa* PPR genes in diverse tissues and under multiple stress treatments

To investigate the expression patterns of *B. rapa* PPR genes, RNA-seq data were obtained from multiple public databases. Raw RNA-seq reads were subjected to quality control and adapter trimming using fastp (version 0.23.4, default parameters) (Chen et al., 2018c). Clean reads were then aligned to the *B. rapa* reference genome using HISAT2 (version 2.2.1) with parameters -p 8 -q -t (Kim et al., 2019). Gene expression quantification was performed with StringTie (version 2.2.1) with parameters -e -B -p 8 -G (Pertea et al., 2016). For differential expression analysis, DESeq2 (version 1.42.1) was applied to datasets with biological replicates (Drought treatment and immunity elicitor treatment RNA-seq data), using a threshold of adjusted P-value ≤ 0.05 and |log_2_(fold change)| ≥ 1 (Love et al., 2014). For datasets without replicates (Heat stress RNA−seq data), edgeR (version 3.42.4) was used with the same significance criteria, according to the edgeR user manual, a dispersion parameter of 0.2 was applied for samples derived from the same genetic background (Robinson et al., 2010).

Gene expression profiles were visualized using the pheatmap R package (Kolde and Kolde, 2015), and expression pattern clusters were identified by k-means clustering.

### 2.6 qRT-PCR validation of expression profiling of *B. rapa* PPR genes in diverse tissues

Chinese cabbage A03 (*B.rapa* ssp. *pekinensis*) was used in this study and grown under natural field conditions in the experimental station of Zhoukou Normal University, Zhoukou, China (33.6° N, 114.6° E). The RNA was obtained from the roots, stems, leaves, flowers and siliques of *B.rapa* ssp. *pekinensis* using the RaPure Plant RNA Kit (Magen Biotechnology Co., Ltd., Guangzhou, China) according to the manufacturer’s protocol. First-strand cDNA synthesis was performed using the PrimeScript RT reagent kit with gDNA Eraser (TaKaRa, RR047A), following the manufacturer’s protocol. The synthesized cDNA served as a template for qRT-PCR, which was performed using the applied Biosystems QuantStudio 1 (Applied Biosystems, Foster City, CA, USA) with 2X M5 HiPer SYBR Premix EsTaq (with Tli RNaseH) (Mei5 Biotechnology, Co., Ltd., Beijing, China). The amplification protocol was as follows: initial denaturation at 95 °C for 30 s, followed by 40 cycles of 95 °C for 5s and 60 °C for 34s. All the primer used in this study were listed in **Supplementary Table 8**. Transcript levels were quantified using the 2^–ΔΔCt method (Livak and Schmittgen, 2001). For each sample, three biological replicates and two technical replicates were analyzed. The mean Ct value of the technical replicates was calculated for subsequent analysis, and *ACTIN* was used as the internal reference gene. Leaf tissue was used as the calibrator sample for ΔΔCt normalization, and relative expression levels were calculated as 2^–ΔΔCt. Differences in tissue−specific expression were assessed by one−way ANOVA followed by Fisher’s LSD post−hoc comparisons (α = 0.05) using R.

### 2.7 RNA editing sites identification

To begin, all RNA-seq reads were mapped to the chloroplast (*B. rapa* var. *purpuraria*, NCBI reference number OP729397.1) (Zhou et al., 2024) and mitochondria (*B. rapa* var. *parachinensis*, NCBI reference number PX776524.1) (Liu et al., 2026) reference genome using HISAT2 (version 2.2.1) with parameters -p 8 -q -t (Kim et al., 2019). Variants/SNPs were then detected and VCF files were generated using bcftools (version 1.6) with parameter --max-idepth 1000000 (Danecek et al., 2021). By integrating SNP-calling outputs with genome annotation files, RNA editing sites and their associated information were identified using REDO (version 1.0) with default parameters. REDO reduces false positives by applying a series of rule-based and statistical filters, including quality control (DP4 = 0; no mapping quality filter is applied for VCF files generated by samtools and bcftools), read depth (total depth <4, alternative depth <3, or total depth <0.2 × adjacent average depth), alternative allele proportion (alternative proportion <0.1 or adjusted proportion <0.1 after error correction), multiple alternative alleles (retaining only single alternative alleles), variant distance (<3 bp), splice junction proximity (<2 bp), indels, likelihood ratio testing (LLR <10), and Fisher’s exact test (P >0.01). In addition, a complex model based on experimentally validated RNA editing sites and codon characteristics further refines candidates using RNA editing type, alternative allele proportion, amino acid changes, codon position, and hydrophobic/hydrophilic alterations (Wu et al., 2018).

RNA editing efficiency was defined as the proportion of edited transcripts relative to the total number of transcripts covering each site. Only RNA editing sites detected in all samples were retained for visualization using the R package pheatmap.

## 3 Results

### 3.1 Identification of PPR genes in *B. rapa* genome

**Figure 1:**
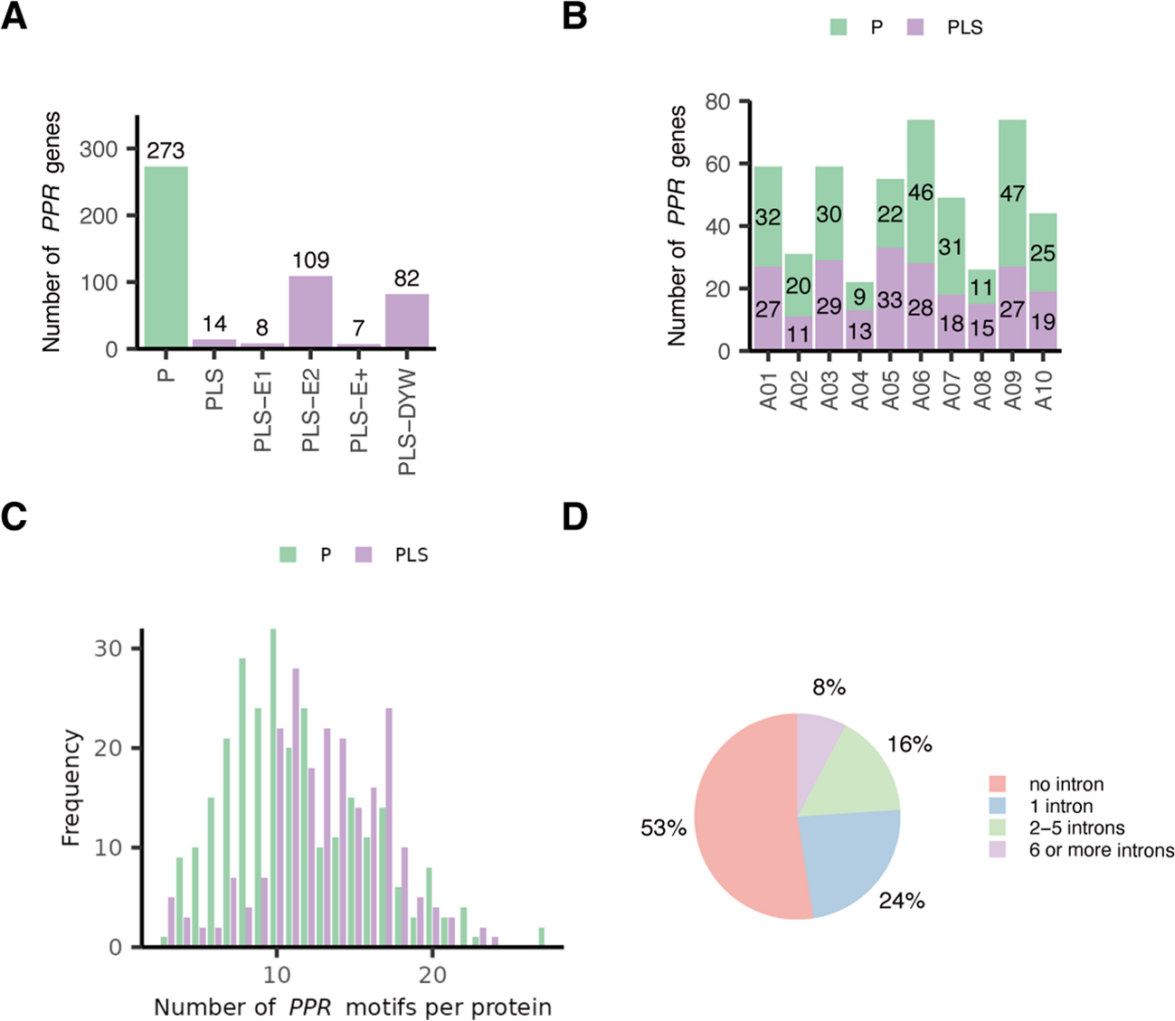
Number of PPR genes and intron distribution in *B. rapa*. **(A)** Distribution of PPR genes among different subgroups, including P, PLS, PLS-E1, PLS-E2, PLS-E+, and PLS-DYW. **(B)** Chromosomal distribution of P-type and PLS-type PPR genes across the 10 chromosomes of *B. rapa*. **(C)** Distribution of motif counts per PPR protein in the P-type and PLS-type subfamilies. **(D)** Proportion of intron numbers per PPR gene in *B. rapa*.

A total of 493 PPR genes were identified in the *B. rapa* genome (**Supplementary Table 1**), comprising 273 P-type and 220 PLS-type members (**Figure 1A**). The PLS-type proteins were further divided into five subgroups: PLS (14), PLS-E1 (8), PLS-E2 (109), PLS-E+ (7), and PLS-DYW (82) (**Figure 1A**). These genes were distributed across all 10 chromosomes of *B. rapa*, although their abundance varied considerably. Chromosomes A06 and A09 contained the highest numbers of PPR genes, whereas chromosome A04 harbored the fewest (**Figure 1B**). Notably, PLS-type proteins exhibited a higher average number of PPR motifs compared with P-type proteins (**Figure 1C**), suggesting greater structural complexity. Analysis of gene structure revealed that more than half (53%) of the PPR genes lacked introns, while 24% contained a single intron. Genes with two to five introns accounted for 16%, whereas only a small proportion (8%) possessed six or more introns (**Figure 1D**). Comprehensive information for all identified PPR genes, including protein sequences and related features, is provided in **Supplementary Table 1**.

### 3.2 Subcellular localization of PPR proteins and function enrichment

**Figure 2:**
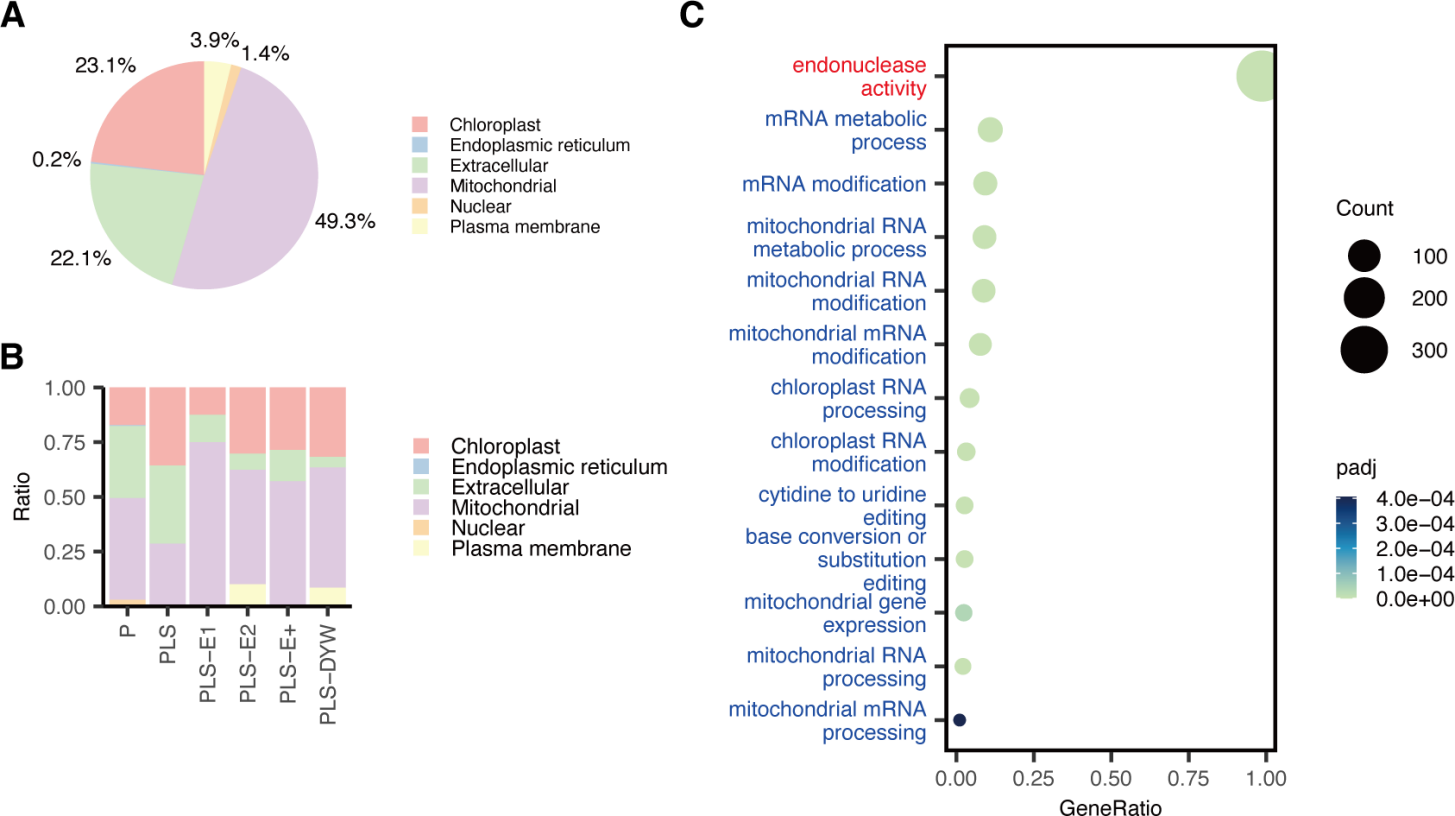
Subcellular localization and functional enrichment of *B. rapa* PPR genes. **(A)** Pie chart showing predicted subcellular localization based on ProtComp. **(B)** Subcellular localization distribution of each PPR subgroup predicted by ProtComp. **(C)** GO enrichment analysis of 493 PPR genes, where blue and red indicate biological processes and molecular functions, respectively.

Given that PPR proteins predominantly function in mitochondria or plastids, their subcellular localization was predicted using ProtComp v9.0. Nearly half (49%) of the PPR proteins were predicted to localize to mitochondria, while 23% were assigned to chloroplasts (**Figure 2A**).

When classified by subfamily, nearly half (47%) of the P-type proteins were predicted to localize to mitochondria, while approximately 17% were assigned to chloroplasts. In contrast, PLS-type proteins exhibited a more even distribution between chloroplasts (36%) and mitochondria (29%). Within the PLS subgroup, PLS-E1 proteins showed a strong bias toward mitochondrial localization (75%), whereas only a small fraction (13%) was predicted to localize to chloroplasts. The PLS-E2, PLS-E+, and PLS-DYW subgroups displayed broadly similar patterns, with approximately half of the proteins predicted to localize to mitochondria and 30% to plastids. (**Figure 2B**; **Supplementary Table 2**).

Consistent with these localization patterns, Gene Ontology (GO) enrichment analysis revealed that PPR genes were significantly enriched in biological processes associated with organellar RNA metabolism, including RNA processing, RNA modification, and cytidine-to-uridine (C-to-U) RNA editing (**Figure 2C**).

### 3.3 Phylogenetic and syntenic analysis of *B. rapa* PPR proteins

**Figure 3:**
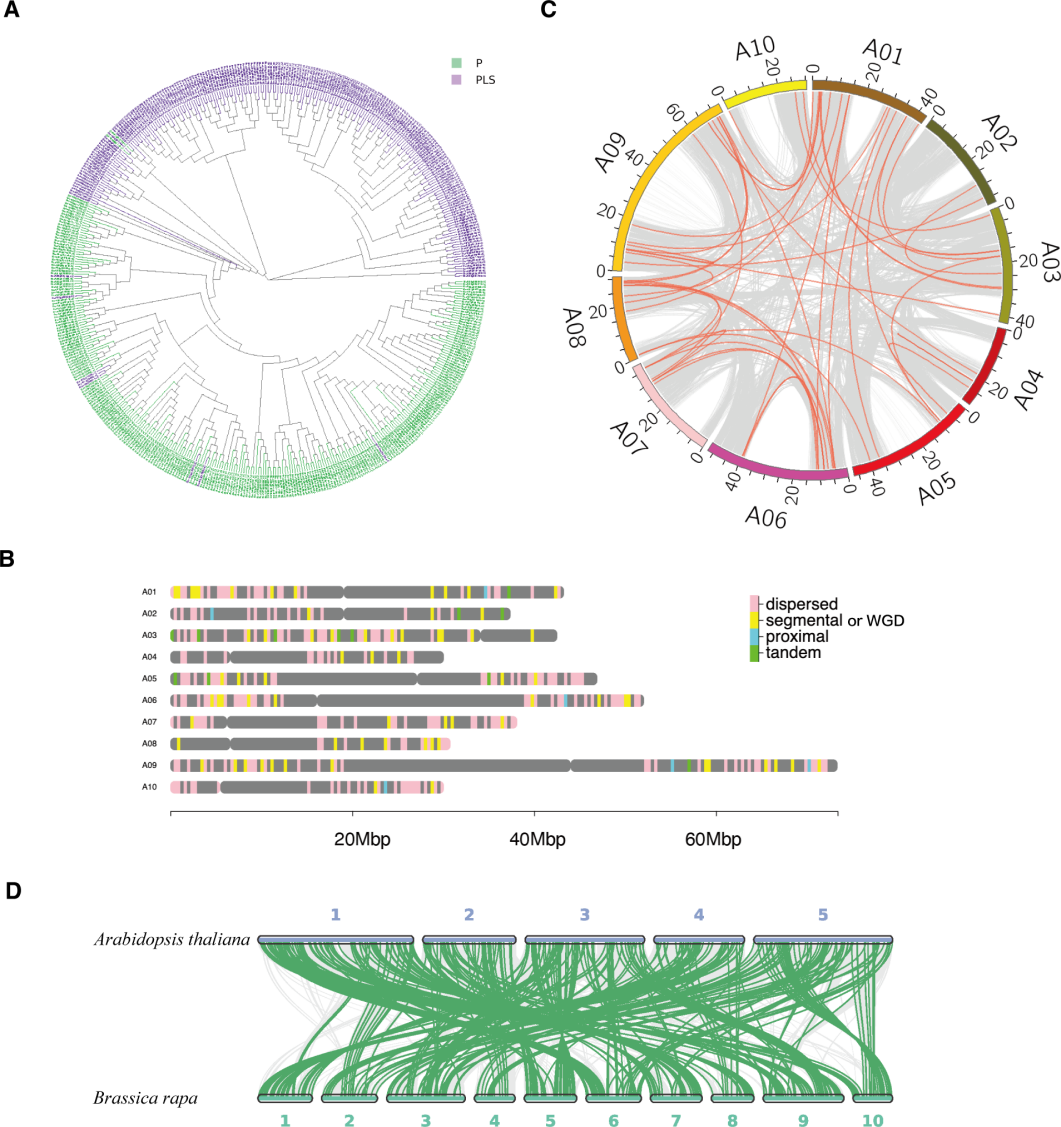
Phylogenetic and synteny analyses of PPR genes in *B. rapa*. **(A)** Phylogenetic tree of 493 PPR genes, with the P subfamily shown in green and the PLS subfamily in purple. **(B)** Distribution of duplicated PPR genes across the 10 chromosomes of *B. rapa*, including dispersed, tandem, whole-genome duplication (WGD)/segmental, and proximal duplications. **(C)** Interchromosomal syntenic relationships of PPR genes in *B. rapa*. Grey lines indicate all syntenic blocks in the genome, while red lines highlight PPR gene pairs. **(D)** Collinearity analysis of PPR genes from *Arabidopsis* and *B. rapa.* Gray lines represent collinear blocks between the *Arabidopsis* and *B. rapa* genomes, while green lines represent the collinearity of PPR gene pairs between the two species.

A phylogenetic tree was constructed using the neighbor-joining (NJ) method based on the full-length amino acid sequences of 493 PPR proteins from *B. rapa*. The resulting tree clearly resolved two major clades corresponding to the P and PLS subfamilies (**Figure 3A**). Notably, eight PLS-type members (BraA08g033700.4C, BraA03g056530.4C, BraA03g018370.4C, BraA01g005750.4C, BraA09g056010.4C, BraA05g025870.4C, BraA04g016860.4C and BraA03g007250.4C) clustered within the P subfamily, whereas two P-type members (BraA01g018480.4C and BraA04g002110.4C) grouped with the PLS subfamily. This inconsistency between phylogenetic clustering and structural classification has also been reported in other species, such as poplar (Xing et al., 2018), where certain PPR proteins exhibit discordant motif composition and evolutionary placement. Such discordance may reflect domain rearrangement or evolutionary convergence within the PPR family.

Given that *B. rapa* has undergone a whole-genome triplication (WGT) event during its evolutionary history (Wang et al., 2011), gene duplication patterns of PPR genes were further investigated using MCScanX. All PPR genes were classified into five duplication categories: dispersed, tandem, whole-genome duplication (WGD)/segmental and proximal, and singleton.

Dispersed duplications constituted the predominant category, accounting for 69% (339/493) of all PPR genes. In addition, 109 genes were derived from WGD or segmental duplications, while 22 and 20 genes derived from tandem and proximal duplications, respectively. Only three PPR genes were identified as singletons (**Figure 3B**; **Supplementary Table 1**).

Collinearity analysis identified 654 syntenic blocks and 20,268 collinear gene pairs across the *B. rapa* genome, among which 71 gene pairs involved PPR genes (**Figure 3C**).

Given that both *Arabidopsis* and *Brassica rapa* belong to the Brassicaceae family, we performed a comparative analysis of PPR proteins between the two species. A total of 420 orthologous gene pairs were identified between *Arabidopsis* and *B. rapa* (**Figure 3D**). *Arabidopsis* has 450 PPR proteins (O’Toole et al., 2008), the presence of 420 orthologous pairs suggests a conserved core set of PPR genes that has been maintained since their divergence from a common ancestor.

### 3.4 Expression patterns of PPR genes in different tissues of *B. rapa*

Gene expression profiles can provide important insights into gene function. To investigate the expression patterns of PPR genes in *B. rapa* across different tissues and organs, transcript abundance was quantified using TPM values (**Supplementary Table 3**). Among the 493 identified PPR genes, 11 genes showed no detectable expression in any of the sampled tissues. This may be due to extremely low expression levels, tissue-specific expression not captured in the dataset, or transcriptional silencing under the sampled conditions. These genes were therefore excluded from subsequent expression pattern analyses.

**Figure 4:**
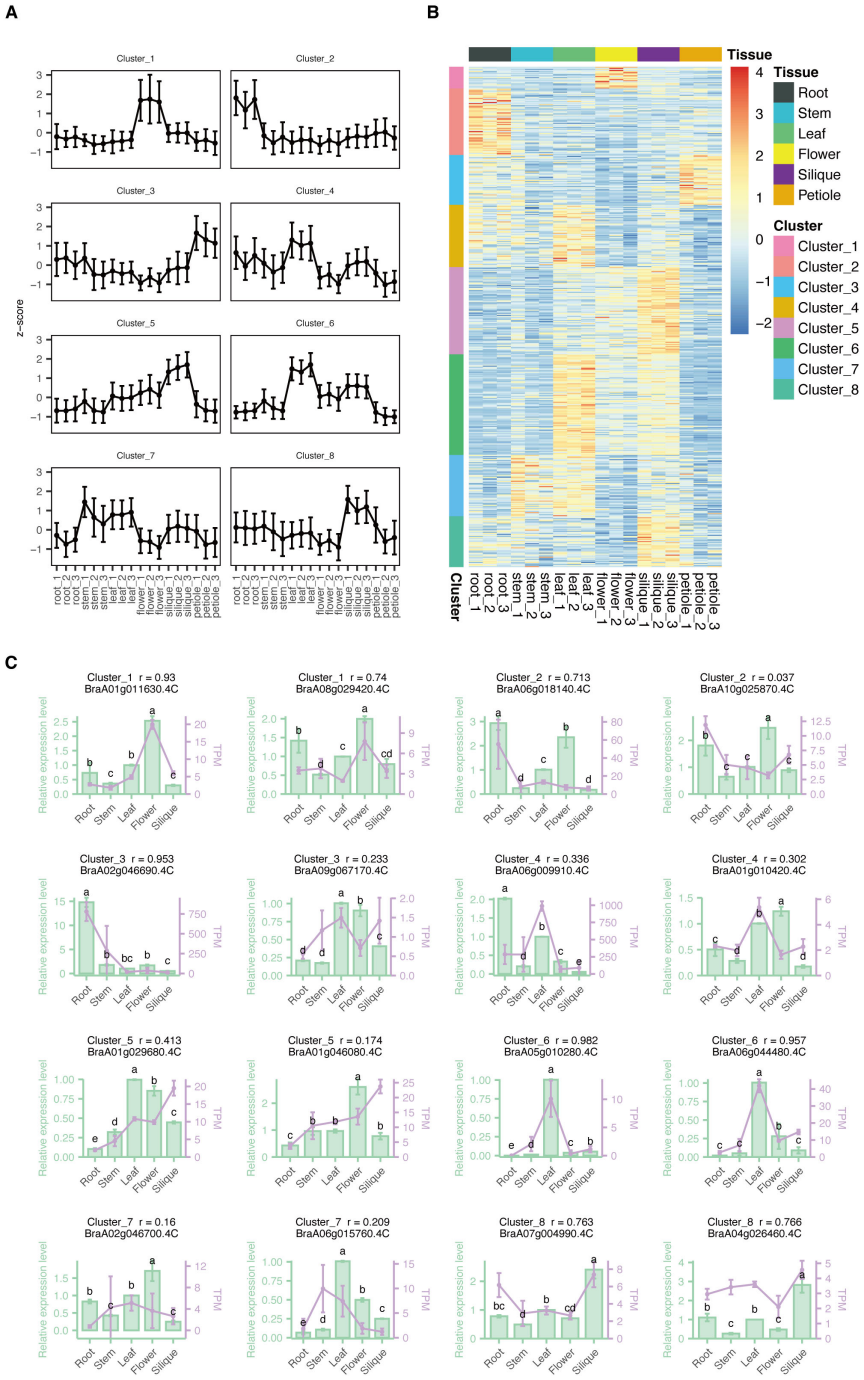
Tissue-specific expression patterns of PPR genes in *B. rapa*. **(A)** K-means clustering identified eight distinct expression profiles among the 493 PPR genes in *B. rapa*. **(B)** Heatmap illustrating the expression patterns of the eight clusters. **(C)** Expression patterns of 16 selected PPR genes analyzed by qRT-PCR, with the *ACTIN* gene as an internal control, leaf as calibrator sample. The bar plot indicates relative expression levels (left y−axis), and the line plot represents TPM values from RNA−seq data (right y−axis). Data are shown as means ± SD from three biological replicates. In the title of each sub plot, r showed Pearson correlation coefficients between relative expression and TPM values. Different lowercase letters above the bars indicate significant differences in relative expression between tissues (LSD, α = 0.05).

The remaining 482 PPR genes were subjected to k-means clustering based on their normalized expression profiles and were grouped into eight distinct clusters (**Figures 4A, B**), each exhibiting characteristic tissue-specific expression patterns. Specifically, Cluster_1 genes were predominantly expressed in flowers, whereas Cluster_2 genes showed preferential expression in roots. Cluster_3 and Cluster_4 genes were mainly expressed in petioles and leaves, respectively. Cluster_5 and Cluster_8 were enriched for genes highly expressed in siliques, while Cluster_6 genes exhibited high expression in leaves with moderate expression in siliques. Cluster_7 genes exhibited elevated expression in stems and leaves. The observed tissue-specific expression patterns indicate potential functional specialization of PPR genes, possibly reflecting distinct roles in organellar gene regulation across developmental contexts (**Figure 4C**).

We selected two representative genes from each cluster for qRT-PCR validation in roots, stems, leaves, flowers and siliques of *B.rapa* ssp. *Pekinensis*, which differs from the genetic background used in the RNA-seq dataset (*B. rapa* ssp. *chinensis*). Overall, the qRT-PCR results showed broadly similar tissue-preferential expression trends to the TPM-based RNA-seq profiles. Genes from Cluster_1, Cluster_6, and Cluster_8 exhibited expression patterns showed similar trends to the corresponding TPM-based profiles, with correlation coefficients greater than 0.7. For Cluster_2, Cluster_4, and Cluster_5, qRT-PCR and TPM data showed generally similar expression trends across most tissues, with only a few discrepancies observed in individual tissues. In Cluster_2, *BraA06g018140.4C* and *BraA10g025870.4C* exhibited relatively higher expression levels in flowers than suggested by the TPM data. In Cluster_4, *BraA06g009910.4C* showed elevated expression in roots, whereas *BraA01g010420.4C* exhibited higher expression in flowers. In Cluster_5, expression levels in siliques were lower than those inferred from the RNA-seq profiles (**Figure 4C**; **Supplementary Tables 4**, **5**).

In Cluster_7, *BraA02g046700.4C* showed some discrepancies in expression patterns in roots and flowers compared with TPM results, possibly due to relatively large standard deviations in the TPM estimates. In addition, the expression pattern of *BraA06g015760.4C* in stems differed from the TPM data, whereas expression patterns in the other tissues remained generally similar. In Cluster_3, the expression pattern of *BraA02g046690.4C* was broadly similar to with the TPM-based profile, with a correlation coefficient of 0.953. For *BraA09g067170.4C*, qRT-PCR analysis showed relatively lower expression in stems and higher expression in flowers compared with the TPM data, while the expression patterns in the remaining tissues were generally comparable (**Figure 4C**; **Supplementary Tables 4**, **5**). Because the correlation analysis was based on only five tissue types for each gene, the resulting correlation coefficients should be interpreted cautiously and provide only partial support for broadly similar tissue-preferential expression trends between the qRT-PCR and RNA-seq datasets. 

These differences may partially result from the distinct genetic backgrounds and experimental conditions between the qRT-PCR materials and the public RNA-seq dataset. Therefore, the qRT-PCR results should be regarded as partial support for broadly similar tissue-preferential expression patterns rather than direct quantitative validation of the RNA-seq data.

### 3.5 Analysis of cis-acting elements in PPR gene promoters of *B. rapa* PPR genes

Cis-acting elements in promoter regions play essential roles in transcriptional regulation and stress responsiveness. To investigate the regulatory potential of PPR genes in *B. rapa*, the 2-kb upstream sequences from the ATG start codon were extracted from the genome and analyzed using the PlantCARE database.

**Figure 5:**
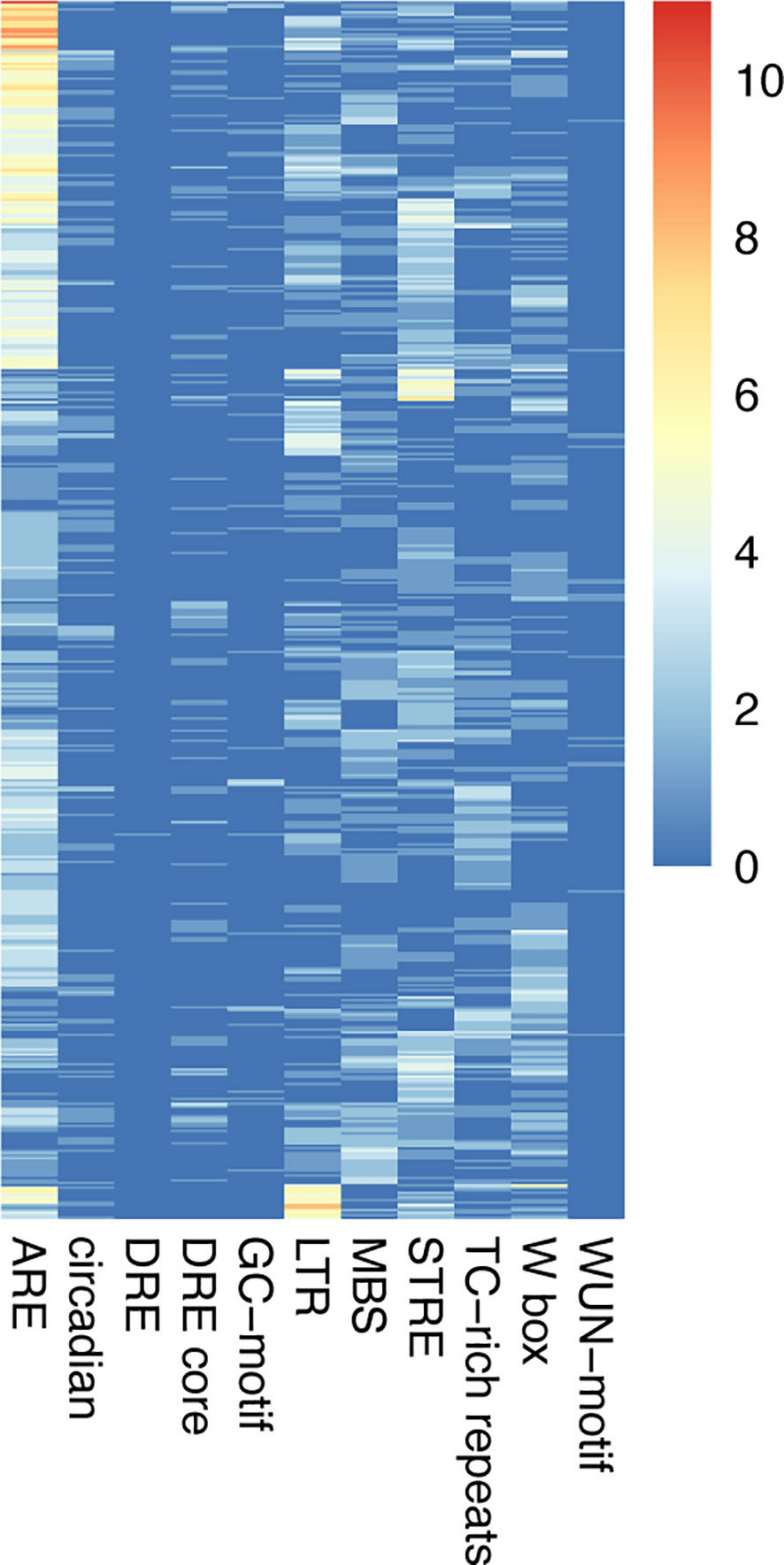
Promoter analysis of 493 PPR genes in *B. rapa*. Heatmap showing the number of cis-elements in the promoters of the 493 PPR genes.

Numerous stress-responsive cis-acting elements were identified in the promoter regions, including ARE, DRE/DRE core, LTR, MBS, STRE, W-box, and WUN-motif (**Figure 5**). These elements are associated with responses to various abiotic stresses, such as drought, low temperature, and anaerobic conditions, as well as biotic and wound-induced stresses. The widespread presence of these elements suggests that PPR genes may play roles in transcriptional regulation under diverse stress conditions, potentially contributing to coordinated stress responses through organellar gene regulation.

### 3.6 Stress-responsive expression patterns of PPR genes in *B. rapa*

Previous studies have suggested that numerous PPR proteins are involved in plant responses to biotic and abiotic stresses, such as those reported in poplar and rice (Chen et al., 2018a; Xing et al., 2018). However, the roles of PPR genes in *B. rapa* under stress conditions remain largely unexplored. To investigate their potential functions in environmental adaptation, we analyzed the expression profiles of *B. rapa* PPR genes under multiple treatments using high-throughput sequencing data, including drought, heat, and immune elicitor treatments.

**Figure 6:**
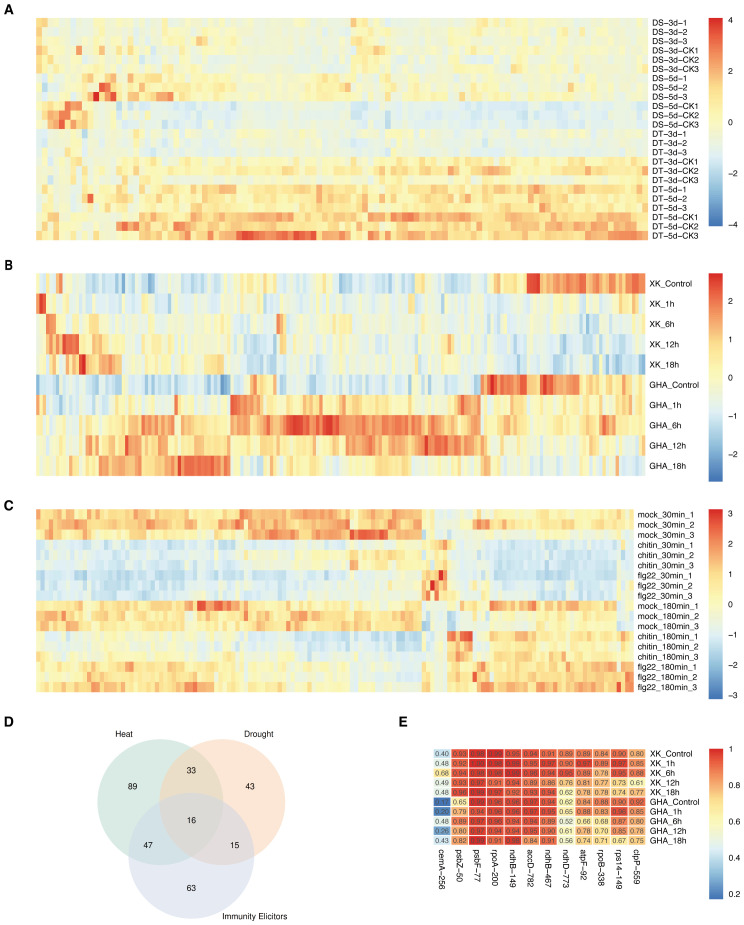
Expression and RNA editing profiles of PPR genes under different stress conditions. **(A)** Expression patterns of PPR genes in *B. rapa* under drought stress. **(B)** Expression patterns of PPR genes in *B. rapa* under heat stress. **(C)** Expression patterns of PPR genes in *B. rapa* under immune elicitor treatment. **(D)** Identification of PPR genes commonly differentially expressed across two or all three stress conditions. **(E)** Chloroplast RNA editing profiles of PPR genes in *B. rapa* under heat stress.

Expression analysis based on publicly available RNA-seq datasets revealed that a substantial number of PPR genes exhibited stress-associated transcriptional changes under different treatment conditions. Specifically, 107, 185, and 141 PPR genes showed altered expression patterns under drought, heat, and immune elicitor treatments, respectively (**Figures 6A–C**; **Supplementary Table 6**). Notably, several PPR genes also displayed altered expression under more than one stress conditions (**Figure 6D**), suggesting potential associations with multiple stress response pathways in *B. rapa*. It should be noted that the heat-treatment dataset lacked biological replication, therefore, the corresponding differential expression results should be considered exploratory rather than statistically definitive.

### 3.7 RNA editing under heat stress in *B. rapa*

To further investigate potential associations between PPR genes and stress-responsive RNA editing, chloroplast and mitochondrial RNA editing events were analyzed using publicly available RNA-seq datasets generated under each condition. However, only the heat-treatment datasets provided sufficient sequencing depth to support reliable RNA editing detection. Therefore, subsequent analyses focused on editing sites consistently detected across all ten heat-treatment samples. However, the chloroplast and mitochondrial reference genomes used in this study differ from the organellar genomic backgrounds of the heat-treatment datasets. In addition, the RNA editing detection pipeline used in this study could not distinguish genomic SNPs from authentic RNA editing events. Therefore, sites showing consistently near-100% editing efficiencies across samples may potentially reflect underlying genomic polymorphisms rather than bona fide RNA editing events. In contrast, sites exhibiting variable editing efficiencies among samples are more likely to represent genuine RNA editing events (**Figure 6E**; **Supplementary Figure 1**; **Supplementary Table 7**).

Notably, the editing efficiencies of several chloroplast transcripts, including *cemA-256*, *psbZ-50* and *ndhD-773*, were generally higher in the heat-resistant line (XK) than in the heat-sensitive line (GHA). These observations suggest a potential association between differential RNA editing patterns and heat-stress responses in *B. rapa* (**Figure 6E**). For *psbZ-50*, the editing efficiency increased from 0.65 to 0.89 after 6 h of heat treatment in GHA, whereas it remained consistently above 0.90 in XK. In contrast, the editing efficiency of *ndhD-773* in XK decreased after 12 h of heat stress and eventually reached a level comparable to that in GHA by 18 h of heat stress, suggesting that sustained high editing at this site may be important for heat tolerance. In addition, the editing efficiencies of *atpF-92*, *rpoB-338*, *rps14-149*, and *clpP-559* declined after 12 h and 18 h of heat treatment in both lines, likely reflecting a general reduction in chloroplast activity under prolonged stress (**Figure 6E**). These observations indicate that chloroplast RNA editing patterns may undergo dynamic changes during heat stress; however, the biological significance of these editing changes remains to be experimentally validated.

Similarly, mitochondrial RNA editing events were also analyzed in heat-treated datasets. Compared with chloroplasts, editing efficiencies were generally consistent and showed only slight variation. Most editing sites showed high efficiencies (~1.0) across treatments, except for *ccb-924*, *ccb-1160*, *nad4-1101*, *nad3-344*, *nad3-349*, *rps3-1464*, and *nad1-725*. *ccb-924* and *ccb-1160* exhibited consistently lower efficiencies, with a gradual decrease under prolonged heat stress in the heat-resistant line (XK). *nad4–1101* decreased from 1.0 to 0.88 and 0.79 after 12 h and 18 h of treatment in XK, respectively. In contrast, *nad3–344* and *nad3–349* increased from ~0.8 to >0.9 following heat treatment in heat-sensitive line (GHA). *rps3–1464* increased in both XK and GHA and remained stable thereafter. For *nad1-725*, XK maintained relatively stable efficiency (0.8–0.9), whereas GHA showed fluctuation (0.83–1.0–0.67–0.84 across time points) (**Supplementary Figure 1**).

Collectively, these results indicate that organellar RNA editing patterns may exhibit site-specific and stress-associated variation under heat treatment. However, because the heat-treatment dataset lacks biological replicates, the observed RNA editing changes should be regarded as descriptive observations rather than statistically validated differences. Furthermore, the functional significance of these RNA editing changes and their potential relationships with PPR proteins remain to be experimentally validated.

## 4 Discussion

PPR proteins are key regulators of organellar gene expression in plants. In this study, we identified 493 PPR genes in *B. rapa* (**Figure 1**; **Supplementary Table 1**), reflecting substantial expansion of this gene family, likely driven by the whole-genome triplication (WGT) event. Duplication analysis indicated that dispersed duplication, together with WGD/segmental duplication, contributed to the expansion and diversification of PPR genes (**Figures 3B, C**). The phylogenetic analysis revealed two major clades corresponding to the P and PLS subfamilies (**Figure 3A**). Notably, eight PLS-type members were found clustered within the P subfamily, whereas two P-type members grouped with the PLS subfamily. This pattern has been documented in other species, including poplar and tobacco (Xing et al., 2018; Wu et al., 2025). suggesting potential domain rearrangement or evolutionary convergence.

Subcellular localization predictions indicated that approximately 49% of PPR proteins target mitochondria and 23% target chloroplasts (**Figure 2A**), consistent with the well-established roles of PPR proteins in organellar gene expression (Barkan and Small, 2014). The higher proportion of mitochondrial targeting predictions may reflect the greater number of PPR proteins required for processing the larger number of RNA editing sites in the mitochondrial transcriptome compared with chloroplasts (e.g., ~481 mitochondrial sites vs ~35 chloroplast sites in rice) (Takenaka et al., 2013). The Gene Ontology enrichment analysis further supported that PPR genes are significantly associated with RNA processing, RNA modification, and cytidine-to-uridine (C-to-U) RNA editing in both mitochondria and chloroplasts (**Figure 2C**). These results confirmed that PPR proteins function as key regulators of organellar gene expression and are essential for maintaining proper organelle function.

The tissue-specific expression patterns revealed by k-means clustering provide valuable insights into potential functional specialization among *B. rapa* PPR genes (**Figure 4**). The eight distinct expression clusters, each showing preferential expression in particular tissues or organs, suggest that different PPR genes may function in diverse developmental contexts. For example, Genes highly expressed in photosynthetically active tissues, such as leaves, may be associated with chloroplast-related functions, whereas genes preferentially expressed in reproductive organs, including flowers and siliques, may play roles in reproductive development.

There is substantial evidence that PPR genes are essential under stress conditions (Zhang et al., 2020; Lee, 2026). Consistently, multiple stress-responsive cis-acting elements, including DRE (dehydration-responsive), LTR (low-temperature-responsive), STRE (stress-responsive element), and W box (wound-responsive) elements, were identified in the promoters of PPR genes (**Figure 5**), suggesting potential transcriptional regulation under stress conditions. In addition, expression analyses based on publicly available RNA-seq datasets revealed that many PPR genes exhibited stress-associated transcriptional changes under drought, heat, and immune elicitor treatments (**Figures 6A–C**). A substantial proportion of PPR genes were differentially expressed under drought (107 genes), heat (185 genes), and immunity elicitor (141 genes) treatments, with dozens of genes responding to multiple stress conditions (**Figure 6D**). Given that the heat-treatment dataset lacks biological replication, the corresponding differential expression results should be interpreted as exploratory. It should be noted that the drought and immune elicitor datasets were derived from *B. rapa* ssp. *pekinensis* (Illumina NovaSeq 6000), whereas heat stress data were obtained from *B. rapa* ssp. *chinensis* (Illumina HiSeq 2000). As the RNA-seq datasets were generated from different genetic backgrounds and sequencing platforms, the observed differences in the number of differentially expressed genes across treatments may partly reflect genotypic variation and technical bias rather than solely stress-specific responses. Nevertheless, the overlapping PPR genes identified across multiple stress conditions provide candidate genes for future investigation of stress-responsive organellar regulation in *B. rapa*. Further studies using uniform genetic backgrounds and experimental validation will be necessary to assess the potential roles of these candidate PPR genes in environmental adaptation.

These results extend previous observations in other plant species (Chen et al., 2018a; Xing et al., 2018) and suggesting that PPR proteins may be associated with stress-responsive regulation of organellar gene expression. Together, these results indicate that PPR genes may be involved in complex transcriptional networks related to organellar function under environmental stress. However, it should be noted that the proposed roles of PPR genes in stress adaptation in this study are primarily inferred from stress-associated expression patterns derived from publicly available RNA-seq datasets, and therefore remain putative and require further experimental validation.

RNA editing analysis further provided insights into potential associations between PPR genes and stress-responsive organellar regulation (Zhang et al., 2023a). It has been well established that RNA editing efficiency is altered under stress conditions (Xiong et al., 2022; Hu et al., 2024). In this study, dynamic and site-specific changes in chloroplast RNA editing were observed under heat stress (**Figure 6E**). Higher editing efficiencies at specific sites, including *cemA*, *psbZ*, and *ndhD*, in the heat-tolerant line suggest potential differences in stress-associated RNA editing patterns between the two genetic backgrounds. Among these sites, the sustained high editing level of *ndhD-773* in the tolerant line, followed by a decline under prolonged stress, highlights its potential importance in early heat response. Given that *ndhD* encodes a subunit of the chloroplast NDH complex involved in photosynthetic electron transport, altered editing efficiency at this site may potentially affect chloroplast cyclic electron transport and energy balance under heat stress. In contrast, the general decline in editing efficiencies at multiple sites, including *atpF*, *rpoB*, *rps14*, and *clpP*, under prolonged heat stress likely reflects a broader impairment of chloroplast activity.

Collectively, these observations indicate that RNA editing responses are site-specific and dynamically regulated, with certain editing events contributing to stress tolerance, while others are more susceptible to stress-induced disruption. Given that PPR proteins are key factors mediating site-specific RNA editing, these findings suggest potential associations between PPR-mediated RNA editing and stress-responsive organellar regulation in *B. rapa*. However, it should be noted that our RNA editing analysis were limited to heat stress datasets due to insufficient sequencing depth in other treatment datasets. In addition, the observed changes in RNA editing efficiencies represent correlative observations rather than direct causal evidence. Furthermore, a limitation of this study is that the stress-associated changes in RNA editing efficiency identified here remain to be experimentally validated.

Future studies involving qRT-PCR validation, RNA editing efficiency assays under stress conditions, and genetic characterization will be necessary to confirm the functional roles of candidate PPR genes in stress adaptation. Nevertheless, this study provides a valuable resource for further investigation of PPR protein functions in organellar gene regulation and plant stress responses in *B. rapa*.

## 5 Conclusion

Our study provides a comprehensive overview of the PPR gene family in *B. rapa* and highlights their evolutionary expansion, structural diversity, expression patterns, and potential associations with stress-responsive organellar regulation. Integrative analyses of publicly available transcriptomic datasets identified tissue-preferential expression patterns, stress-associated transcriptional changes, and dynamic variation in organellar RNA editing under heat stress. These findings provide candidate PPR genes and RNA editing sites for future investigation and offer a valuable resource for further studies on PPR protein functions in organellar gene expression and plant stress responses *B. rapa*.

## Data Availability

Publicly available datasets were analyzed in this study. Tissue expression data were downloaded from the Genome Sequence Archive (GSA) of the BIG Data Center (https://ngdc.cncb.ac.cn/gsa/) under accession number CRA010488. The genetic background is *B. rapa ssp. chinensis*, and RNA sequencing was performed using the Illumina HiSeq 4000 platform (Liu et al., 2023). RNA-seq data for drought treatment were retrieved from the same database under accession number CRA009052. These data were derived from *B.rapa* ssp. *pekinensis* genotypes, including the drought_tolerant genotype 14S837 (DT) and the drought_sensitive genotype 88S148 (DS), with sequencing conducted on the Illumina NovaSeq 6000 platform (Chen et al., 2024). Heat stress RNA_seq data were downloaded from NCBI under accession number SRP064703; the genetic background is *B. rapa* ssp. *chinensis*, and sequencing was performed on the Illumina HiSeq 2000 platform (Wang et al., 2016). Immunity elicitor treatment data were obtained from NCBI under accession number GSE150746; the genetic background is *B. rapa* ssp. *pekinensis*, and sequencing was carried out on the Illumina NovaSeq 6000 platform (Kim et al., 2020).

## Supplementary material

Supplementary Figure 1 Mitochondrial RNA editing profiles of PPR genes in *B. rapa* under heat stress.
