# Supplementary figures and images for: Genome-wide characterization of the PPR gene family and its potential roles in stress responses and chloroplast RNA editing in Brassica rapa
Source: Front Plant Sci. 2026 Jun 15;17:1860005. doi: 10.3389/fpls.2026.1860005 (PMC13311020; doi:10.3389/fpls.2026.1860005)

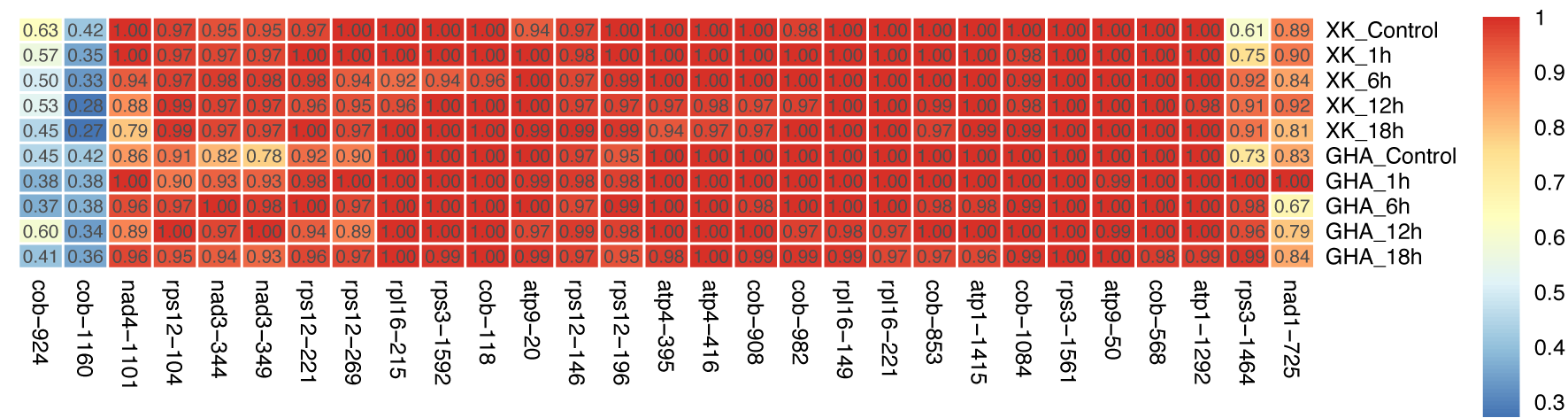

Supplement: Supplementary Figure 1 — Mitochondrial RNA editing profiles of PPR genes in B. rapa under heat stress. [file Image1.tif]
